# Supplementary material for: A Likelihood Approach for Real-Time Calibration of Stochastic Compartmental Epidemic Models
Source: PLoS Comput Biol. 2017 Jan 17;13(1):e1005257. doi: 10.1371/journal.pcbi.1005257 (PMC5240920; doi:10.1371/journal.pcbi.1005257)
Supplement: S1 File — (TAR.GZ) [file pcbi.1005257.s014.tar.gz › HSPH_Online-SI-Revision/MSS10/n1-severe/n1-severe_NEW_table0.pdf]

1	R0	{1.51081, 13.7078, 1.}	{1.23922, 3.01076, 0.03}	{{1.22544, 2.5364, 0.}, {1.25474, 3.26445, 0.}}	{17.9766, 78.0362, 97.}	inf	54.	20.3203	{7.80233, 33.2969}	62.4721	0.503749	newly	34.	40.9	{11., 71.}	52.8235	0.235075	Reff	1.05303	0.905423	{0.8795, 0.931268}	14.0178	ar	656.	358.32	{272., 461.}	45.378
2	R0	{1.73182, 11.3873, 3.}	{1.30094, 3.64095, 0.05}	{{1.22544, 3.07157, 0.}, {1.44762, 4.00571, 0.}}	{24.8803, 68.0262, 98.3333}	inf	80.	31.3692	{10.6023, 52.6266}	60.7885	0.477657	newly	53.	53.78	{22., 88.}	41.3208	0.209733	Reff	1.15166	0.928271	{0.875777, 1.0266}	19.3972	ar	653.	418.72	{311., 526.}	35.8775
3	R0	{1.50449, 10.3474, 0.1}	{1.26544, 3.37295, 0.08}	{{1.22544, 2.5364, 0.}, {1.31748, 3.98513, 0.}}	{15.8894, 68.478, 174.}	inf	60.	24.1704	{7.85523, 39.0785}	60.3814	0.47931	newly	43.	47.2	{14., 82.}	49.0233	0.23705	Reff	1.08474	0.957396	{0.921672, 1.00922}	12.0102	ar	651.	370.61	{256., 497.}	43.0707
4	R0	{1.63493, 9.1955, 0.1}	{2.33007, 17.2089, 0.81}	{{2.04669, 13.0262, 0.}, {2.46477, 18.9032, 1.}}	{42.518, 87.1443, 748.}	inf	87.	196.45	{129.479, 232.211}	125.805	0.344114	newly	59.	82.12	{69., 97.}	39.4237	0.140472	Reff	1.05126	1.2365	{1.18473, 1.2836}	17.6206	ar	663.	861.84	{800., 900.}	29.991
5	R0	{1.53822, 8.05553, 0.1}	{1.75918, 11.5624, 0.81}	{{1.67732, 9.44073, 0.}, {1.93882, 15.8893, 2.}}	{15.08, 46.3329, 828.}	inf	55.	114.449	{81.2126, 164.916}	110.388	0.310986	newly	40.	69.33	{49., 87.}	73.975	0.231588	Reff	1.01061	1.04739	{1.00555, 1.0843}	4.32172	ar	621.	709.95	{638., 793.}	16.3205
6	R0	{1.69374, 17.0793, 0.1}	{1.64165, 16.0794, 0.53}	{{1.514, 12.483, 0.}, {1.75588, 19.2972, 1.}}	{6.96434, 19.6198, 524.}	inf	97.	73.5535	{46.4838, 97.5072}	25.1075	0.140886	newly	40.	34.34	{26., 42.}	17.55	0.0876058	Reff	1.25676	1.25454	{1.17412, 1.32777}	5.16125	ar	662.	657.7	{542., 749.}	10.1299
7	R0	{1.42179, 11.8031, 2.}	{1.59131, 13.6025, 1.84}	{{1.28596, 3.98513, 0.}, {1.66055, 15.2961, 2.}}	{14.2307, 31.647, 12.}	inf	57.	83.232	{35.8429, 108.804}	62.729	0.244666	newly	30.	42.29	{27., 55.}	47.7667	0.172057	Reff	0.963974	1.03382	{0.931608, 1.06822}	8.4219	ar	517.	627.43	{473., 722.}	26.8221
8	R0	{1.46683, 6.95875, 0.1}	{2.52828, 17.1857, 0.76}	{{2.11132, 12.644, 0.}, {2.76795, 19.6041, 1.}}	{72.39, 147.814, 712.}	inf	92.	246.891	{178.406, 296.205}	168.853	0.420888	newly	97.	98.25	{84., 112.}	9.02062	0.0389383	Reff	0.815557	1.00014	{0.954428, 1.04831}	22.633	ar	653.	890.58	{823., 938.}	36.6769
9	R0	{1.34525, 11.505, 1.}	{1.83932, 19.7824, 1.98}	{{1.84468, 19.9256, 2.}, {1.84468, 19.9256, 2.}}	{36.7271, 71.9461, 98.}	inf	47.	111.378	{98.1533, 121.641}	136.973	0.373372	newly	31.	41.16	{34., 50.}	33.2258	0.120185	Reff	1.01701	1.27203	{1.25465, 1.29739}	25.0756	ar	532.	743.55	{701., 776.}	39.765
10	R0	{1.77958, 14.372, 0.1}	{1.92267, 13.7279, 0.}	{{1.63746, 10.1332, 0.}, {2.42205, 19.4475, 0.}}	{15.2279, 22.0627, 100.}	inf	110.	163.602	{91.1216, 234.591}	56.9229	0.179663	newly	47.	77.8	{61., 95.}	65.5319	0.211892	Reff	0.948516	0.905785	{0.871869, 0.946427}	4.75824	ar	671.	775.89	{657., 880.}	17.0626
11	R0	{1.60334, 19.9548, 1.}	{1.53319, 11.5347, 0.03}	{{1.53432, 11.4771, 0.}, {1.53432, 11.4771, 0.}}	{4.3754, 42.1957, 101.}	inf	94.	74.6432	{61.9453, 85.9294}	20.652	0.104028	newly	31.	46.57	{37., 57.}	50.5484	0.171549	Reff	1.08867	1.07072	{1.05341, 1.08985}	1.84406	ar	617.	605.32	{541., 657.}	5.79903
12	R0	{1.68623, 7.58585, 0.1}	{2.71895, 16.9604, 0.96}	{{1.95791, 9.82151, 1.}, {2.93488, 18.8445, 1.}}	{61.2585, 123.658, 870.}	inf	74.	255.529	{138.759, 299.665}	245.309	0.521706	newly	59.	102.16	{86., 118.}	73.1525	0.235529	Reff	0.881898	0.907058	{0.859, 0.948256}	3.98199	ar	640.	899.1	{765., 943.}	40.4844
13	R0	{1.82689, 18.0022, 1.}	{1.43837, 7.02215, 0.18}	{{1.38815, 6.00596, 0.}, {1.44485, 6.71734, 0.}}	{23.0721, 61.192, 102.}	inf	136.	46.2375	{26.6736, 61.8145}	66.0018	0.500995	newly	49.	45.48	{29., 62.}	22.2857	0.110195	Reff	1.10344	0.99546	{0.952587, 1.01483}	11.9038	ar	673.	506.58	{421., 583.}	26.4398
14	R0	{1.75911, 9.16883, 0.1}	{2.3768, 16.4199, 0.86}	{{2.11132, 12.644, 0.}, {2.49092, 17.9391, 1.}}	{37.9261, 86.319, 790.}	inf	93.	191.09	{155.425, 222.501}	114.025	0.344616	newly	75.	84.38	{69., 100.}	18.16	0.0756914	Reff	1.16629	1.32914	{1.2293, 1.40853}	15.8874	ar	661.	853.	{821., 907.}	33.5582
15	R0	{1.46168, 11.3859, 2.}	{1.70149, 12.5196, 1.99}	{{1.56965, 10.7075, 2.}, {1.73739, 13.0272, 2.}}	{16.4063, 12.9046, 0.5}	inf	70.	103.268	{81.1155, 120.614}	47.6586	0.164595	newly	36.	55.77	{44., 67.}	54.9167	0.185124	Reff	0.809771	0.885148	{0.846234, 0.916502}	9.30844	ar	556.	697.81	{621., 749.}	25.5054
16	R0	{1.53577, 11.7581, 1.}	{1.70699, 10.5009, 1.25}	{{1.44485, 6.00596, 0.}, {2.1968, 19.9579, 3.}}	{15.6769, 28.1206, 107.}	inf	94.	91.4524	{42.8299, 153.288}	34.1123	0.169938	newly	55.	62.7	{48., 78.}	22.2182	0.0914631	Reff	1.05507	1.16797	{1.03523, 1.3182}	11.4739	ar	625.	672.54	{516., 825.}	18.1152
17	R0	{1.70698, 9.72924, 0.1}	{1.83594, 8.76401, 0.81}	{{1.44762, 3.83841, 0.}, {2.73517, 19.6025, 2.}}	{24.2543, 62.1456, 828.}	inf	101.	107.743	{27.5043, 233.136}	71.9049	0.339606	newly	63.	77.09	{45., 102.}	35.8889	0.141034	Reff	0.913234	0.931579	{0.834362, 1.08497}	8.23588	ar	675.	668.17	{486., 905.}	23.0741
18	R0	{1.5973, 14.931, 0.1}	{1.83408, 14.8847, 2.13}	{{1.73739, 13.0272, 2.}, {1.95859, 19.3194, 3.}}	{15.3658, 13.578, 2032.}	inf	53.	86.7548	{69.2743, 106.954}	66.1212	0.216677	newly	23.	44.78	{32., 55.}	94.6957	0.279142	Reff	1.28583	1.41266	{1.33453, 1.51413}	10.268	ar	632.	733.	{664., 804.}	17.038
19	R0	{1.87468, 17.004, 1.}	{1.67521, 15.4614, 2.94}	{{1.68078, 15.6632, 3.}, {1.68078, 15.6632, 3.}}	{10.64, 9.07223, 196.}	inf	112.	123.906	{113.458, 136.134}	13.9726	0.0613366	newly	45.	54.1	{44., 64.}	22.2222	0.0852699	Reff	0.987956	0.86247	{0.845039, 0.882512}	12.7016	ar	692.	710.33	{674., 748.}	5.02746
20	R0	{1.84715, 12.531, 0.1}	{2.2194, 19.1478, 0.}	{{2.2194, 19.1478, 0.}, {2.2194, 19.1478, 0.}}	{20.1527, 52.8032, 100.}	inf	90.	208.902	{196.74, 220.215}	132.113	0.365292	newly	55.	72.99	{62., 84.}	32.7091	0.120457	Reff	0.936505	0.861345	{0.835924, 0.888194}	8.02559	ar	661.	860.28	{836., 879.}	30.1483
21	R0	{1.75938, 13.5959, 2.}	{1.32917, 4.72639, 0.18}	{{1.22544, 3.07157, 0.}, {1.44485, 6.71734, 1.}}	{24.8692, 66.0627, 93.}	inf	92.	33.4568	{10.9323, 54.221}	66.1283	0.536826	newly	50.	45.86	{21., 68.}	34.24	0.18634	Reff	1.1911	0.97559	{0.91742, 1.03307}	18.1004	ar	620.	432.94	{291., 551.}	31.0161
22	R0	{1.63456, 10.7469, 0.1}	{1.47937, 3.99256, 0.03}	{{1.44762, 3.83841, 0.}, {1.53501, 4.06661, 0.}}	{9.49455, 62.8492, 128.}	inf	85.	42.8649	{25.0237, 62.3678}	49.5708	0.323478	newly	61.	61.72	{38., 88.}	26.8525	0.123579	Reff	0.81728	0.801977	{0.768664, 0.831927}	2.95178	ar	654.	558.07	{487., 613.}	14.6682
23	R0	{1.62702, 19.1392, 1.}	{1.26329, 3.30428, 0.04}	{{1.22544, 2.5364, 0.}, {1.28596, 3.98513, 0.}}	{22.3555, 82.7356, 96.}	inf	97.	21.5783	{7.20886, 36.1643}	77.7544	0.737402	newly	37.	40.29	{10., 68.}	49.3784	0.255661	Reff	1.02014	0.88731	{0.856229, 0.907824}	13.1742	ar	600.	384.47	{288., 476.}	35.9217
24	R0	{1.70466, 12.1202, 2.}	{1.35773, 3.40521, 0.01}	{{1.22544, 2.5364, 0.}, {1.47608, 4.06661, 0.}}	{20.3516, 71.9047, 99.5}	inf	70.	21.9381	{6.047, 37.7031}	68.6598	0.574139	newly	32.	36.89	{9., 64.}	47.7813	0.217723	Reff	0.946086	0.81842	{0.744478, 0.899144}	13.4942	ar	613.	468.2	{394., 549.}	23.6215
25	R0	{1.61735, 11.3169, 0.1}	{1.46031, 4.40214, 0.03}	{{1.44485, 3.83841, 0.}, {1.47608, 6.00596, 0.}}	{9.70971, 61.1012, 128.}	inf	103.	36.775	{17.2793, 54.6076}	64.2962	0.482877	newly	62.	51.05	{29., 77.}	28.5323	0.162487	Reff	0.826466	0.842889	{0.809421, 0.872187}	3.09934	ar	681.	524.62	{456., 583.}	22.9633
26	R0	{1.56977, 12.3035, 2.}	{1.30431, 4.388, 0.3}	{{1.22544, 3.07157, 0.}, {1.3618, 4.98002, 1.}}	{18.3113, 65.3989, 86.}	inf	78.	30.2028	{9.60763, 49.3772}	65.9372	0.532925	newly	42.	42.85	{18., 64.}	36.8333	0.180258	Reff	1.03605	0.92066	{0.870755, 0.966223}	11.9326	ar	594.	408.54	{294., 518.}	33.3603
27	R0	{1.46317, 5.8298, 0.1}	{1.53931, 5.22595, 0.38}	{{1.39162, 1.89883, 0.}, {1.82711, 13.6186, 2.}}	{7.14521, 55.7829, 442.}	inf	64.	59.1933	{22.592, 128.836}	46.7838	0.23267	newly	64.	76.1	{42., 117.}	36.125	0.145706	Reff	0.847175	0.894231	{0.83647, 0.940876}	6.14203	ar	586.	584.51	{465., 740.}	13.5068
28	R0	{1.70002, 14.9806, 1.}	{2.25832, 18.6985, 1.99}	{{2.25962, 18.788, 2.}, {2.29738, 19.0355, 2.}}	{32.8405, 25.5565, 99.}	inf	110.	178.168	{155.919, 201.91}	62.1219	0.207437	newly	42.	70.65	{58., 82.}	68.3571	0.222335	Reff	1.16281	1.38994	{1.35411, 1.42929}	19.5324	ar	652.	855.46	{822., 889.}	31.2055
29	R0	{1.52729, 16.1014, 3.}	{1.61102, 13.6394, 1.49}	{{1.42801, 9.02643, 1.}, {1.8189, 18.8228, 2.}}	{12.847, 31.5162, 50.3333}	inf	86.	92.37	{36.2268, 153.059}	58.9458	0.272235	newly	44.	42.	{25., 57.}	23.4545	0.112268	Reff	0.925538	0.95573	{0.906863, 1.00432}	4.22432	ar	592.	628.96	{466., 783.}	21.2331
30	R0	{1.72795, 13.5635, 1.}	{1.44345, 5.98968, 0.07}	{{1.44485, 6.00596, 0.}, {1.44485, 6.00596, 0.}}	{16.4648, 55.8397, 97.}	inf	92.	31.6534	{19.0684, 42.9088}	65.5941	0.481295	newly	43.	33.08	{20., 47.}	28.4651	0.158109	Reff	0.865703	0.810271	{0.792732, 0.828981}	6.40317	ar	668.	522.59	{470., 575.}	21.768
31	R0	{1.51591, 18.1544, 3.}	{1.61927, 18.2749, 3.73}	{{1.44476, 10.1243, 2.}, {1.64587, 19.5316, 4.}}	{8.03881, 12.3842, 33.}	inf	59.	103.38	{58.1027, 118.698}	78.309	0.246039	newly	16.	40.94	{33., 50.}	155.875	0.402473	Reff	1.1051	1.1073	{1.07203, 1.12977}	1.55654	ar	541.	674.96	{581., 733.}	25.5712
32	R0	{1.4282, 10.3534, 0.1}	{1.88543, 16.5692, 1.01}	{{1.75663, 14.6725, 1.}, {2.11476, 19.5406, 1.}}	{32.0145, 60.0362, 910.}	inf	38.	99.8717	{85.1278, 110.908}	162.82	0.417258	newly	21.	44.74	{35., 53.}	113.048	0.322954	Reff	1.1397	1.38757	{1.29951, 1.5357}	21.7486	ar	521.	752.53	{701., 812.}	44.4395
33	R0	{1.56693, 11.3972, 0.1}	{1.82794, 10.9967, 1.51}	{{1.44485, 6.00596, 0.}, {2.04423, 14.1631, 2.}}	{20.2964, 27.5329, 1454.}	inf	107.	123.156	{50.7149, 175.48}	44.9513	0.195431	newly	62.	75.66	{53., 98.}	29.2258	0.112374	Reff	0.965229	1.08479	{0.963587, 1.18154}	12.6076	ar	695.	718.04	{530., 833.}	16.2245
34	R0	{1.50675, 19.8823, 1.}	{1.45451, 13.4988, 3.96}	{{1.45653, 13.6021, 4.}, {1.45653, 13.6021, 4.}}	{3.46699, 32.1067, 298.}	inf	68.	59.5777	{50.0683, 69.3661}	13.7567	0.0680124	newly	22.	29.93	{24., 37.}	37.4091	0.133143	Reff	0.90405	0.884878	{0.871753, 0.899149}	2.15226	ar	587.	568.09	{520., 614.}	5.75639
35	R0	{1.72889, 7.76068, 0.1}	{2.0315, 13.0709, 1.76}	{{1.71337, 8.80249, 1.}, {2.35866, 16.9062, 2.}}	{19.0322, 69.2597, 1660.}	inf	77.	153.051	{95.9755, 206.555}	100.122	0.28494	newly	62.	82.1	{69., 94.}	32.9355	0.120741	Reff	1.04771	1.06492	{0.983049, 1.16927}	6.07807	ar	650.	781.32	{657., 879.}	21.2246
36	R0	{1.62644, 4.12303, 0.1}	{1.39451, 1.94126, 0.06}	{{1.39162, 1.89883, 0.}, {1.39162, 1.89883, 0.}}	{14.2602, 52.9166, 148.}	inf	43.	31.4369	{9.84226, 54.6148}	38.16	0.267356	newly	57.	61.64	{19., 98.}	44.0702	0.229168	Reff	0.769306	0.675688	{0.642075, 0.704381}	12.1692	ar	652.	564.45	{508., 619.}	13.4279
37	R0	{1.70878, 15.8072, 1.}	{1.40594, 7.44663, 0.15}	{{1.39882, 5.7404, 0.}, {1.44485, 8.26611, 1.}}	{17.7226, 52.8909, 89.}	inf	84.	32.1769	{21.3937, 42.1246}	61.6942	0.432549	newly	36.	28.26	{17., 38.}	29.8333	0.160803	Reff	0.915906	0.826451	{0.807756, 0.852728}	9.76679	ar	674.	502.52	{438., 554.}	25.4421
38	R0	{1.58967, 6.80568, 0.1}	{1.91212, 11.8158, 1.67}	{{1.71337, 8.80249, 1.}, {2.01902, 13.4708, 2.}}	{20.2842, 73.6172, 1570.}	inf	73.	143.447	{90.9514, 184.288}	96.7289	0.277042	newly	63.	80.3	{67., 96.}	29.0794	0.108117	Reff	0.86637	0.902078	{0.871647, 0.934965}	4.17744	ar	610.	769.29	{680., 839.}	26.1754
39	R0	{1.344, 8.19498, 0.1}	{1.89679, 18.2848, 1.95}	{{1.91377, 18.7084, 2.}, {1.91377, 18.7084, 2.}}	{41.3933, 126.185, 1854.}	inf	39.	146.853	{133.243, 163.736}	276.811	0.568066	newly	36.	57.73	{48., 69.}	60.5278	0.200789	Reff	0.928704	1.10408	{1.0781, 1.13707}	19.1446	ar	551.	772.17	{741., 815.}	41.2214
40	R0	{1.61509, 7.90825, 0.1}	{1.24814, 3.02182, 0.}	{{1.22544, 2.5364, 0.}, {1.25474, 3.26445, 0.}}	{22.7201, 61.7891, 100.}	inf	48.	24.1533	{8.81579, 39.1597}	50.3082	0.369023	newly	41.	45.75	{17., 76.}	44.2683	0.20564	Reff	1.04173	0.83477	{0.811865, 0.855613}	19.8672	ar	581.	409.84	{341., 472.}	29.4871
41	R0	{1.60017, 5.90605, 0.1}	{2.33851, 14.9088, 0.}	{{2.11292, 12.9719, 0.}, {2.93978, 19.9817, 0.}}	{46.1413, 152.433, 100.}	inf	76.	196.409	{150.451, 279.558}	158.433	0.400336	newly	76.	87.86	{75., 102.}	17.2895	0.067711	Reff	0.832088	0.9208	{0.87472, 0.968469}	10.7109	ar	661.	853.73	{793., 926.}	29.1573
42	R0	{1.64436, 15.1337, 1.}	{1.7288, 17.1409, 4.42}	{{1.58389, 10.8293, 3.}, {1.8129, 19.688, 5.}}	{8.38801, 29.7228, 348.}	inf	74.	104.507	{61.279, 132.663}	50.0493	0.17795	newly	39.	43.65	{33., 53.}	18.3333	0.0728605	Reff	1.11323	1.11462	{1.06973, 1.1517}	2.65657	ar	640.	695.55	{590., 771.}	13.9828
43	R0	{1.48917, 11.917, 0.1}	{1.59349, 13.1157, 0.}	{{1.59546, 13.1646, 0.}, {1.59546, 13.1646, 0.}}	{7.12679, 10.6711, 100.}	inf	72.	83.3245	{71.4601, 94.4427}	17.2742	0.068037	newly	36.	43.65	{35., 54.}	22.75	0.0853501	Reff	0.927753	0.974641	{0.956841, 0.992579}	5.11744	ar	551.	635.82	{580., 682.}	15.6189
44	R0	{1.65577, 7.89317, 1.}	{1.78264, 10.8391, 0.64}	{{1.67732, 9.44073, 0.}, {1.81647, 11.5667, 1.}}	{7.91382, 38.3075, 56.}	inf	65.	108.43	{83.1165, 132.394}	67.1815	0.213495	newly	60.	70.13	{56., 86.}	20.0833	0.0766022	Reff	1.09446	1.09834	{1.05617, 1.13676}	2.45764	ar	576.	715.96	{639., 773.}	24.3194
45	R0	{1.6909, 12.3275, 0.1}	{1.80306, 13.2024, 1.63}	{{1.54207, 9.08517, 1.}, {2.05052, 16.7932, 2.}}	{13.6321, 27.6841, 1536.}	inf	82.	110.58	{58.1263, 155.124}	49.9424	0.181023	newly	47.	56.56	{39., 70.}	26.383	0.101708	Reff	0.968886	0.974062	{0.889586, 1.03787}	5.76981	ar	675.	711.63	{567., 811.}	13.6963
46	R0	{1.62351, 16.0698, 2.}	{1.43073, 11.8525, 1.98}	{{1.34829, 9.33397, 2.}, {1.44709, 12.3743, 2.}}	{11.8742, 26.2436, 1.}	inf	58.	55.6698	{38.3143, 70.6872}	16.9011	0.0850373	newly	28.	33.43	{24., 44.}	26.75	0.103831	Reff	1.25173	1.10606	{1.07108, 1.1285}	11.6371	ar	591.	531.45	{449., 611.}	12.2352
47	R0	{1.52372, 4.38599, 0.1}	{2.63427, 15.6712, 0.94}	{{2.47514, 14.5291, 1.}, {2.90165, 18.0404, 1.}}	{72.9469, 257.552, 856.}	inf	71.	251.097	{218.771, 281.66}	254.536	0.545369	newly	95.	111.49	{94., 126.}	18.7053	0.0730939	Reff	0.888329	1.05391	{0.978805, 1.11571}	18.6397	ar	618.	901.95	{879., 937.}	46.0728
48	R0	{1.44306, 11.788, 1.}	{1.46828, 9.3398, 1.59}	{{1.34436, 6.25107, 0.}, {1.70573, 16.6329, 3.}}	{8.94922, 44.1465, 125.}	inf	70.	60.9638	{21.3955, 131.58}	60.6138	0.31492	newly	36.	39.19	{22., 57.}	31.8611	0.135798	Reff	0.883153	0.905019	{0.870642, 0.947482}	3.2063	ar	534.	541.55	{412., 733.}	19.2753
49	R0	{1.63624, 11.3893, 1.}	{2.39238, 17.868, 1.83}	{{2.1255, 13.6009, 1.}, {2.48947, 19.255, 2.}}	{46.6822, 60.9111, 87.}	inf	91.	226.581	{187.031, 257.547}	152.499	0.402722	newly	55.	87.07	{77., 100.}	59.6545	0.201966	Reff	0.927748	1.02418	{0.974545, 1.06703}	10.466	ar	643.	872.61	{848., 909.}	36.5086
50	R0	{1.46113, 11.1322, 1.}	{1.88158, 17.7832, 1.86}	{{1.91377, 17.7016, 2.}, {1.93087, 18.8819, 2.}}	{30.021, 67.7284, 98.}	inf	63.	114.663	{97.8593, 132.969}	88.8083	0.285323	newly	52.	46.97	{36., 57.}	14.8654	0.0745002	Reff	1.09731	1.31231	{1.28784, 1.35691}	20.3503	ar	501.	753.43	{697., 817.}	52.7685
